# Risk factors for cement leakage and nomogram for predicting the intradiscal cement leakage after the vertebra augmented surgery

**DOI:** 10.1186/s12891-020-03810-4

**Published:** 2020-11-30

**Authors:** Tian-yu Zhang, Pei-xun Zhang, Feng Xue, Dian-ying Zhang, Bao-guo Jiang

**Affiliations:** 1 Department of Traumatic Orthopaedics, Peking University People’s Hospital, No.11 South Avenue, Xi Zhi Men, Xicheng District, Beijing, 100044 China; 2 Institute of Trauma and Nerve Regeneration, Peking University People’s Hospital, Beijing, 100044 China; 3 Department of Orthopaedics, Peking University Binhai Hospital, Tianjin, 300450 China

**Keywords:** Cement leakage, Risk factors, Nomogram, Vertebral augmentation, Vertebral compression fracture

## Abstract

**Background:**

Vertebral augmentation is the first-line treatment for the osteoporosis vertebral compression fractures. Bone cement leakage is the most common complication of this surgery. This study aims to assess the risk factors for different types of cement leakage and provides a nomogram for predicting the cement intradiscal leakage.

**Methods:**

We retrospectively reviewed 268 patients who underwent vertebral augmentation procedure between January 2015 and March 2019. The cement leakage risk factors were evaluated by univariate analysis. Different types of cement leakage risk factors were identified by the stepwise logistic analysis. We provided a nomogram for predicting the cement intradiscal leakage and used the concordance index to assess the prediction ability.

**Results:**

A total of 295 levels of vertebrae were included, with a leakage rate of 32.5%. Univariate analysis showed delayed surgery and lower vertebral compression ratio were the independent risk factors of cement leakage. The stepwise logistic analysis revealed percutaneous vertebroplasty was a risk factor in vein cement leakage; delayed surgery, preoperative compression ratio, and upper endplate disruption were in intradiscal cement leakage; age, preoperative fracture severity, and intravertebral vacuum cleft were in perivertebral soft tissue cement leakage; no factor was in spinal canal cement leakage. The nomogram for intradiscal cement leakage had a precise prediction ability with an original concordance index of 0.75.

**Conclusions:**

Delayed surgery and more vertebral compression increase the risk of cement leakage. Different types of cement leakage have different risk factors. We provided a nomogram for precise predicting the intradiscal cement leakage.

## Background

Osteoporosis vertebral compression fractures (OVCFs) are the most common type of osteoporosis fracture. First-line approach for pain relief and kyphosis correction of OVCFs is vertebral augmentation, including percutaneous vertebroplasty (PVP) and percutaneous kyphoplasty (PKP) [1, 2]. Though the technique of operation has developed, surgical complications still remain great concern.

The cement leakage ranks first among the complications of vertebral augmentation [3], defined as the presence of any extra-vertebral cement. It includes four main types: vein leakage, intradiscal leakage, perivertebral soft tissue leakage, and spinal canal leakage [4]. Though most leakage is asymptomatic, severe outcomes could happen, such as pulmonary emboli [5, 6] caused by cement vein leakage and nerve compression caused by spinal canal cement leakage [7, 8]. These are catastrophic complications. Moreover, the intradiscal leakage increases the risk of adjacent vertebral fractures [9–11] leading to recurrence of pain. Hence, it is vital to find the risk factors for the leakage to instruct us in predicting and preventing it.

The purpose of this study is to evaluate the independent risk factors of different types of cement leakage and provide a nomogram for predicting the intradiscal cement leakage after PKP and PVP.

## Methods

### Selection criteria

This study is a retrospective cohort study. We retrospectively reviewed 507 vertebral compression fracture patients in our medical center between January 2015 and June 2019. The patients need to meet the inclusion criteria as follows: (1) symptomatic OVCFs; (2) treated with unilateral PVP or PKP; (3) magnetic resonance imaging (MRI) revealed the fresh vertebral fracture related to the back pain. The exclusion criteria were as follows: (1) fracture caused by tumor or infection; (2) patients underwent the vertebral fusion surgery at the augmented level; (3) patients with radiating pain. Finally, 268 patients with 295 levels of vertebrae were included.

### Factors measurement

According to the preoperative X-ray, cortical disruption was divided into four types: the disruption of the upper endplate, the lower endplate, the anterior wall, and the posterior wall. The time before surgery was divided into the early stage for less than 30 days and delayed stage for more than 30 days between trauma and surgery. Preoperative fracture severity was classified into grade 1: mild (< 25% collapse) and grade 2: moderate to severe (> 25%) [12]. Preoperative MRI was used to identify preoperative intravertebral vacuum cleft (IVC). The performance of the IVC was an area of hypointensity on T1-weighted, hyper or hypointensity signal on T2-weighted and T2-fat suppression images with “double line” on the margin of the area [13].

**Fig. 1:**
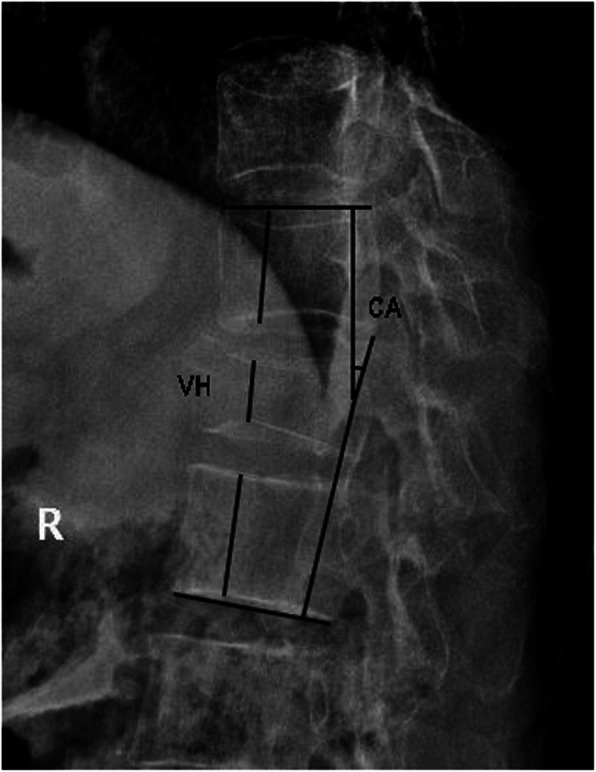
The measurement method of the vertebral height (VH) and the Cobb angle (CA)

The radiologic parameters were vertebral height (VH) and Cobb angle (CA) measured from the X-ray. We chose the vertebra’s maximal compression point for VH measurement and the corresponding point of upper and lower vertebra [14] (Fig. 1). The CA was measured between the superior endplate of the vertebra above the augmented vertebra and the lower endplate of the vertebra below the augmented vertebra [15] (Fig. 1). The vertebral compression ratio was the ratio of preoperative fractured vertebral VH to the average of upper and lower VH.

We assessed the risk factors that potentially influenced the cement leakage, including the age, gender, time before surgery, preoperative fracture severity, preoperative CA, preoperative vertebral compression ratio, preoperative IVC, cement volume, surgery method (PKP or PVP), upper endplate disruption, lower endplate disruption, posterior wall disruption, and anterior wall disruption.

### Surgical procedures

All surgeries were performed by a group of experienced surgeons. Patient lay in a prone position with local anesthesia. Vertebral augmentation approach was unipedicle. After the puncture needle arrived at the anterior 1/3 of the vertebra, “toothpaste-like” cement was injected into it. A balloon was inflated to restore the vertebral height in the PKP before the cement injection. All operations were performed under the guidance of the C-arm machine. The patient was asked to have bed rest for at least 5 h after the surgery and wear the brace for 1 month.

### Outcome measurement

**Fig. 2:**
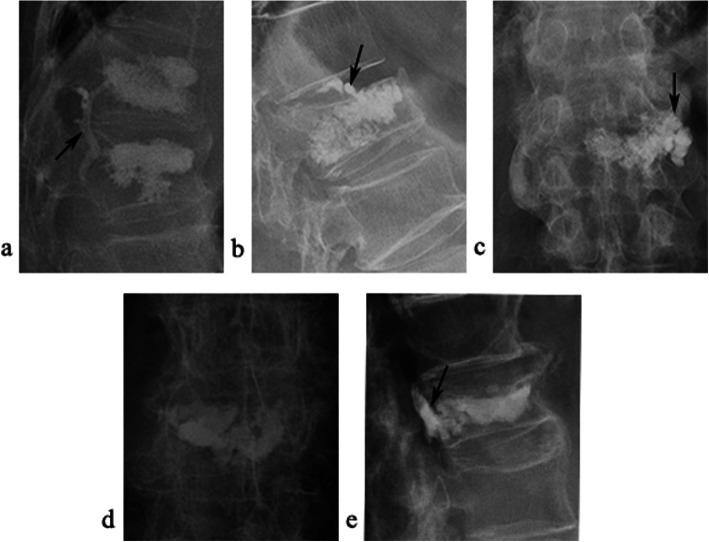
Imaging manifestation of different types vertebral cement leakage. Postoperative X-ray **a** lateral position X-ray reveals the cement vein leakage (arrow) **b** lateral position X-ray reveals the intradiscal cement leakage (arrow) **c** anterior-posterior (AP) position X-ray reveals the cement paravertebral leakage (arrow) **d** and **e** AP and lateral position X-ray revealed the cement spinal canal leakage (arrow)

As the postoperative CT was not necessary for the vertebra augmented [16], we chose the X-ray for assessing the cement leakage. According to the previous studies [3, 17], we classified the cement leakage patterns into: (1) the vein leakage, (2) the perivertebral soft tissue leakage, (3) the spinal canal leakage, (4) the intradiscal leakage (Fig. 2).

### Statistical analysis

Univariate and multivariate analysis was performed using SPSS Ver. 22.0 for Windows (IBM Corp. NY, USA). Univariate analysis was carried out with Chi square tests for dichotomous factors and univariate logistic regression for continuous factors. *P* value < 0.05 was considered as significant difference. Stepwise binary logistic analysis was performed for finding the risk factors of different types cement leakage. 0.05 was the cut-off P value for the statistical difference. The nomogram and the concordance index were provided by R statistical software (R 3.6.3, R Foundation for Statistical Computing, Vienna, Austria).

## Results

We retrospectively reviewed 268 patients (56 males and 212 females) from January 2015 to March 2019. A total of 295 levels of vertebrae were included, and the cement leakage rate was 32.5% (Table 1). 240 (89.6%) patients received one vertebra augmented, 27 (10.1%) patients received 2 vertebrae augmented and 1 (0.4%) patient received 3 vertebrae augmented. The most common cement leakage type was intradiscal leakage (44, 14.9%) followed with perivertebral soft tissue leakage (35, 11.9%), vein leakage (22, 7.5%), and spinal canal leakage (8, 2.7%) (Table 1).

**Table 1:** Characteristics of patients

Number of patients, n (%)	268
Male	56 (20.8)
Female	212 (79.2)
Mean age, y	74.1
Levels of vertebrae, n	295
Cortical disruption, n (%)	
Upper endplate	189 (64.1)
Lower endplate	30 (10.2)
Anterior wall	79 (26.8)
Posterior wall	7 (2.4)
Number of treated vertebrae Per session, n (%)	
1	240 (89.6)
2	27 (10.1)
3	1 (0.4)
Surgery method, n (%)	
PKP	198 (67.1)
PVP	97 (32.9)
Preoperative IVC, n (%)	68 (23.1)
Total cement leakage, n (%)	96 (32.5)
Vein leakage	22 (7.5)
Intradiscal leakage	44 (14.9)
Perivertebral soft tissue leakage	35 (11.9)
Spinal canal leakage	8 (2.7)

*PKP* percutaneous kyphoplasty, *PVP* percutaneous vertebroplasty, *IVC* intravertebral vacuum cleft

Univariate analysis showed that delayed surgery (OR = 1.85, 95% CI = 0.76–2.12, *P* = 0.033) was the risk factor of cement leakage and univariate logistic analysis showed that vertebral compression ratio (OR = 0.23, 95% CI = 0.65–0.83, *P* = 0.025) was the risk factor of cement leakage (Table 2).

**Table 2:** Chi-square tests were performed for dichotomous factors of cement leakage and univariate logistic regression analyses were performed for continuous factors of cement leakage

Preoperative factors (n)	Cement leakage (n,%)	OR (95%CI)	*P* value
Dichotomous factors			
Gender			0.653
Male (65)	23 (35.4)	1	
Female (230)	73 (31.7)	0.85 (0.48–1.52)	
Time before surgery			0.033*
Early stage (218)	63 (28.9)	1	
Delayed stage (77)	33 (42.9)	1.85 (1.08–3.16)	
Preoperative fracture severity			0.104
Grade 1 (132)	36 (27.3)	1	
Grade 2 (163)	60 (36.8)	1.27 (0.76–2.12)	
Preoperative IVC			0.184
No (227)	69 (30.4)	1	
Yes (68)	27 (39.7)	1.51 (0.86–2.65)	
Surgery method			0.428
PKP (198)	61 (30.8)	1	
PVP (97)	35 (36.1)	1.55 (0.94–2.56)	
Continuous factors			
Age (years)		1.02 (0.99–1.05)	0.144
Preoperative CA		1.01 (0.99–1.04)	0.398
Preoperative compression ratio (%)		0.23 (0.65–0.83)	0.025*
Cement volume (ml)		0.99 (0.76–1.28)	0.935

*IVC* intravertebral vacuum cleft, *PKP* percutaneous kyphoplasty, *PVP* percutaneous vertebroplasty, *CA* Cobb angle

*P < 0.05

All factors potentially influencing the cement leakage, as mentioned in the methods section, were included in the stepwise binary logistic analysis. Stepwise binary logistic analysis revealed that PVP (OR = 5.52, 95% CI = 2.05–14.89, *P* = 0.001) was an independent risk factor of vein leakage. Delayed surgery (OR = 2.74, 95% CI = 1.35–5.59, *P* = 0.005), preoperative vertebral compression ratio (OR = 0.13, 95% CI = 0.02–0.84, *P* = 0.032), and upper endplate disruption (OR = 2.74, 95% CI = 1.14–6.56, *P* = 0.024) were risk factors of intradiscal leakage. Age (OR = 1.06, 95% CI = 1.01–1.12, *P* = 0.011), preoperative fracture severity (OR = 2.82, 95% CI = 1.20–6.61, *P* = 0.017), and preoperative IVC (OR = 2.88, 95% CI = 1.33–6.23, *P* = 0.007) were risk factors of perivertebral soft tissue leakage. No risk factor was relevant with the spinal canal leakage (Table 3).

**Table 3:** Results of stepwise logistic analysis for risk factors of four different type leakage patterns

Leakage patterns and risk factors	OR	95%CI	*P* values
Vein leakage			
Surgery method (PVP)	5.52	2.05–14.89	0.001
Intervertebral leakage			
Delayed surgery	2.74	1.35–5.59	0.005
Preoperative compression ratio	0.13	0.02–0.84	0.032
Upper endplate disruption	2.74	1.14–6.56	0.024
Perivertebral soft tissue leakage			
Age	1.06	1.01–1.12	0.011
Preoperative fracture severity	2.82	1.20–6.61	0.017
Preoperative IVC	2.88	1.33–6.23	0.007
Spinal canal leakage			
Preoperative CA	0.93	0.85–1.02	0.119*

*IVC* intravertebral vacuum cleft, *PVP* percutaneous vertebroplasty, *CA* Cobb angle

*P > 0.05

**Fig. 3:**
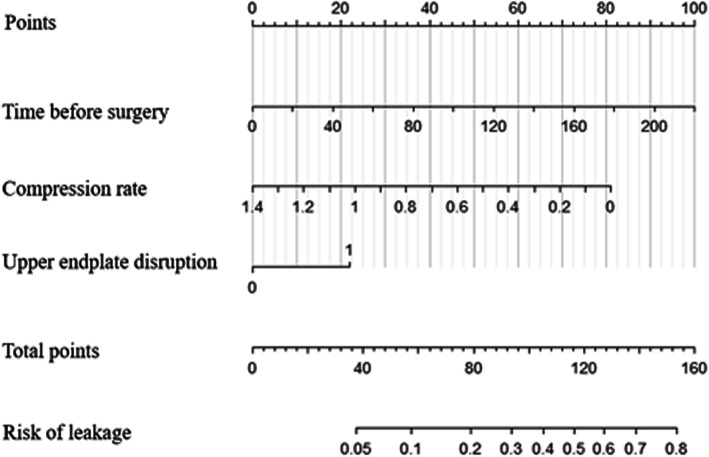
Nomogram for the intradiscal cement leakage. Mark the values at each factor axis, acquire the corresponding points at the points axis, and sum up the points of all factors. Mark the total points on the total point axis and draw a perpendicular line towards the risk of leakage axis. The value on the bottom line gives the probability of the cement leakage. Upper endplate disruption: yes = 1, no = 0

**Fig. 4:**
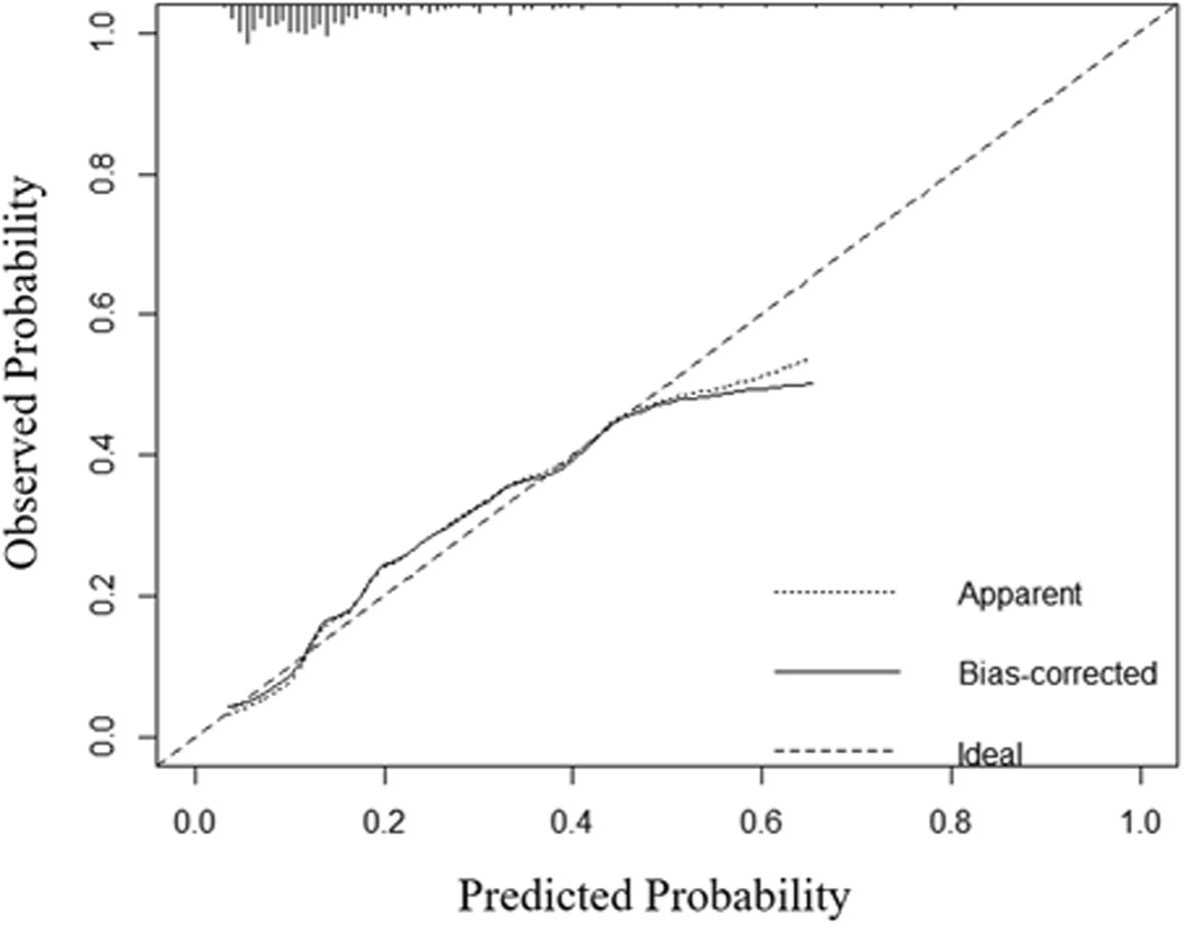
Calibration of the nomogram. The x-axis presents the predicted probability and the y-axis presents the observed probability. The concordance index is 0.75

We depicted the nomogram according to the independent risk factors in the stepwise binary logistic analysis (Fig. 3). To use the nomogram for predicting the risk of intradiscal cement leakage, we can locate the patients values at each factor axis, acquire the corresponding points and sum them up. Then we can locate the sum on total point axis and draw downward to the risk axis to acquire the probability of intradiscal leakage. Nomogram for intradiscal cement leakage had an original concordance index of 0.75 (Fig. 4).

## Discussion

To the best of our knowledge, we firstly provided a nomogram for predicting the cement intradiscal leakage after the vertebral augmentation identified by the postoperative X-ray. Moreover, we assessed the influence of different cortical disruption types on cement leakage.

The upper endplate disruption was the most common type in the vertebral cortical disruption (64.1%) and the second was anterior wall disruption (26.8%). This meets the basic biomechanics rules. When a person falls, the stress is from the top to bottom, and the upper endplate is firstly destroyed. The anterior wall breaks as the stress becomes stronger. Only under huge stress, the lower endplate (10.2%) and posterior wall (2.4%) got involved.

We reported a cement leakage rate of 32.5%. In previous studies, the cement leakage rate identified by postoperative X-ray was 58.2% after PVP [17]. The CT identification cement leakage rate was 76.8–81% [3, 18, 19]. Though CT was more sensitive than X-ray, X-ray was enough for most leakage detection and performed as a proper radiological tool [16]. CT was needed only when spinal canal leakage or neurological symptoms after vertebral augmentation happens.

Delayed surgery was found to be a risk factor of cement leakage. Theoretically, hematoma absorption and fracture line hardening result in higher intravertebral pressure by the time according to bone healing process, and the cement is more likely to leak to the direction of low pressure. Therefore, the cement is more likely to leak in delayed surgery group. However, Guan [20] reported the contrary result. Ren [21] evaluated the freshness of vertebral fracture identified by preoperative MRI, and found no relationship with the cement leakage. This may cause by the different criterion of time point. More vertebral compression was also the risk factor of cement leakage. The vertebral compression ratio was agreed as a risk factor for cement leakage by numerous studies [18, 19, 22, 23]. The more compression of the vertebra always means the more cortical breakage, which results in cement leakage.

We analyzed different types of cement leakage because of their different sequelae.

Cement vein leakage was mostly asymptomatic. However, cement pulmonary embolism might be catastrophic [5]. PVP was the risk factor for the vein leakage (OR = 5.46) comparing to PKP. The inflated balloon provided a cavity for the cement, which reduced the pressure during injection process [24]. As a consequence, the vein cement leakage could be reduced.

Numerous studies showed that intradiscal cement leakage could increase adjacent vertebral fractures [9–11, 25]. Delayed surgery, vertebral compression ratio, and upper endplate breakage were the independent risk factors for intradiscal cement leakage. Delayed surgery may increase the intradiscal cement leakage through the same mechanism mentioned above. The preoperative vertebral compression ratio and cortical disruption were reported as the risk factors in the prior studies [18, 23, 26]. The vertebral compression reduced the volume of the vertebra resulting in higher pressure as less volume of cement injection. The upper endplate disruption increases the risk of the intradiscal cement leakage was first proposed. It may increase the leakage rate by disrupting the vertebral integrity.

Perivertebral soft tissue cement leakage was asymptomatic. However, it increases the cement injection volume and causes the thermal damage to perivertebral soft tissue. Age, preoperative fracture severity, and preoperative IVC were the independent risk factors of cement perivertebral soft tissue leakage. The preoperative fracture severity and preoperative IVC were reported as risk factors for cement leakage previously [18, 27]. Severe fracture and cleft could both produce space for the cement leakage. Age is related to the severity of osteoporosis, and severe osteoporosis leads to the cement leakage. This can explain the age is the risk factor of perivertebral soft tissue cement leakage.

No factor was significantly associated with spinal canal cement leakage. Spinal canal cement leakage seldom leads to neurological injury because the injection would be ceased once the spinal leakage happens under the guidance of C-arm. Therefore, the leakage volume in spinal is always low.

Nomogram is a graphical model in which the probability of the outcome can be calculated. It has been improved to be a feasible model in risk prediction. Zhong [26] provided a nomogram for predicting the intradiscal cement leakage based on postoperative CT. However, CT after the vertebral augmentation was not cost-effective and practical in most of the hospitals for identifying the cement leakage. X-ray predicting cement leakage is more useful for clinical use. Moreover, the nomogram was not calibrated in his study. Hence, we depicted the nomogram based on a postoperative X-ray and performed a calibration.

Our nomogram could provide a precise prediction ability for the intradiscal cement leakage with a concordance index of 0.75. It is composed of the time before surgery, preoperative vertebral compression ratio, and upper endplate disruption. Therefore, the surgeon can acquire the possibility of intradiscal cement leakage before the surgery and make a well preparation for surgery.

The limitation was that this study was a retrospective research, which could bring the selection bias. We did not calculate the ratio of cement volume with the vertebral volume. The cement volume alone could not predict cement leakage [26]. Finally, the cement viscosity has not been assessed. The cement viscosity was regarded as a risk factor influencing the cement leakage [18, 28]. However, it was difficult to objectively evaluate the cement viscosity because multiple factors could influence it, such as surgeon experience, cement property, and mixing method.

## Conclusions

Delayed surgery and more vertebral compression increase the risk of cement leakage of vertebral augmentation. Different types of cement leakage have different risk factors. We provided a nomogram for precise predicting the intradiscal cement leakage.

## Data Availability

The datasets generated and analyzed during the current study are not publicly available due to the data also forms part of an ongoing study but are available from the corresponding author on reasonable request.

## Abbreviations

OVCFs: Osteoporosis vertebral compression fractures
PVP: Percutaneous vertebroplasty
PKP: Percutaneous kyphoplasty
MRI: Magnetic resonance imaging
IVC: Intravertebral vacuum cleft
VH: Vertebral height
CA: Cobb angle
OR: Odds ratios
CI: Confidence interval
